# Supplementary material for: Which resources help young people to prevent and overcome mental distress in deprived urban areas in Latin America? A protocol for a prospective cohort study
Source: BMJ Open. 2021 Sep 13;11(9):e052339. doi: 10.1136/bmjopen-2021-052339 (PMC8438828; doi:10.1136/bmjopen-2021-052339)
Supplement: Supplementary data [file bmjopen-2021-052339supp001.pdf]

Supplementary File 1: Schedule of assessments for the cross-sectional and longitudinal studies

Item measured	Scale/ assessment method	Description of scale	Baseline	6-month follow-up	12-month follow-up
**DEMOGRAPHICS**					
Demographics	Current living situation	Includes variables like gender, age, education level etc	X		X*
	History of depression and anxiety	Participants' and their parents' experiences of mental distress and treatment received	X		X*
**MEASURES OF MENTAL DISTRESS**					
Degree of distress	Patient Health Questionnaire-8 (PHQ-8)	Measures degree of experiencing a list of symptoms associated with depression	X	X	X
	Generalised Anxiety Disorder-7 (GAD-7)	Measures degree of experiencing a list of symptoms associated with anxiety	X	X	X
Drug use	The Alcohol, Smoking and Substance Involvement Screening Test (ASSIST) - adapted	Measures drug use in the lifetime and last 3 months	X		X
	Adapted Teen Addiction Severity Index (T-ASI) - adapted	Measures drug use including alcohol use and severity	X		X
Life events	Adolescent appropriate life events scale - adapted	Captures experience of life events in their lifetime and in the last year or six month respectively	X	X	X
**MEASURES OF RESOURCES**					
General resources	Open question	What participants do when they feel mentally distressed	X		X
Impact of COVID-19	Closed questions	Brief assessment of how COVID-19 has impacted on various activities	X	TBC	TBC
**MEASURES OF PERSONAL RESOURCES**					
Quality of life	Manchester Short Assessment of Quality of Life (MANSA)	Measures perception of how satisfied they are with different aspects of their lives	X		X
Sex life	Closed question	Whether they have had sex with another person in the last month	X		X
Coping style	Child's Coping Strategy Checklist	Measures how individuals deal with problems and stress	X		X
Resilience	Connor-Davidson Resilience Scale (CD-RISC 10)	Measures resilience in response to stressful events, tragedy or trauma	X		X
**MEASURES OF SOCIAL RESOURCES**					
Use of healthcare and other services	Client Service Receipt Inventory (CSRI) - adapted	Measures frequency of use of healthcare and social services	X	X	X
Social support	Scale of Perceived Social Support	Measures perception of social support network	X		X

Social capital	Adapted Social Capital Assessment Tool (ASCAT) - adapted	Asks about perceptions of and engagement with community groups	X		X
Family support	Open questions	Asks which family member they speak to about feelings and emotions, and which family members speak to them about feelings and emotions	X		X
**MEASURES OF ACTIVITIES**					
Sports activity	Open/closed questions	Asks about sports activities including frequency of participation and the nature of these activities	X		X
Arts activity	Open/closed questions	Asks about arts activities including frequency of participation and the nature of these activities	X		X
Internet use	Question 59 from REACH study - adapted	Measures internet use via agreement with a list of statements	X		X

Legend:
X: questionnaire included
X: various items included (e.g. only sociodemographic items that might have changed will be included)*
TBC: to be confirmed depending on the COVID-19 situation in partner countries
