# Supplementary material for: Which resources help young people to prevent and overcome mental distress in deprived urban areas in Latin America? A protocol for a prospective cohort study
Source: BMJ Open. 2021 Sep 13;11(9):e052339. doi: 10.1136/bmjopen-2021-052339 (PMC8438828; doi:10.1136/bmjopen-2021-052339)
Supplement: Supplementary data [file bmjopen-2021-052339supp002.pdf]

Supplementary File 2: Summary of questions that form the ESM assessment

Item measured	Question	Description
Location	Where are you now?	1 item asking participants to state their location. Participants choose from predefined categories or select "other" and provide more detail
Activity	What are you doing now?<br>Time spent doing this activity since last "beep" (notification)?	1 open question asking participants what they are doing (main activity)<br>1 item asking the participants to indicate time spent doing this activity since the last notification
Company	Who are you doing this activity with?	1 item asking participants to state who they are doing their main activity with. Participants choose from predefined categories or select "other" and provide more detail.
Affect	How are you feeling now?	1 item asks participants to rate how they are feeling on a 7-point Likert scale ranging from 1 ("extremely unhappy") to 7 ("extremely happy")
Arousal	How nervous or relaxed are you now?	1 item asking participants to rate how nervous/relaxed they are feeling on a 7-point Likert scale ranging from 1 "very nervous" to 7 "very relaxed"
