# Supplementary material for: Which resources help young people to prevent and overcome mental distress in deprived urban areas in Latin America? A protocol for a prospective cohort study
Source: BMJ Open. 2021 Sep 13;11(9):e052339. doi: 10.1136/bmjopen-2021-052339 (PMC8438828; doi:10.1136/bmjopen-2021-052339)
Supplement: Author's manuscript [file bmjopen-2021-052339.draft_revisions.pdf]

BMJ Open

BMJ Open is committed to open peer review. As part of this commitment we make the peer review history of every article we publish publicly available.

When an article is published we post the peer reviewers' comments and the authors' responses online. We also post the versions of the paper that were used during peer review. These are the versions that the peer review comments apply to.

The versions of the paper that follow are the versions that were submitted during the peer review process. They are not the versions of record or the final published versions. They should not be cited or distributed as the published version of this manuscript.

BMJ Open is an open access journal and the full, final, typeset and author-corrected version of record of the manuscript is available on our site with no access controls, subscription charges or pay-per-view fees (http://bmjopen.bmj.com).

If you have any questions on BMJ Open's open peer review process please email info.bmjopen@bmj.com

BMJ Open

Which resources help young people to prevent and overcome mental distress? A programme of observational studies in deprived urban areas in Latin America

Journal:	*BMJ Open*
Manuscript ID	bmjopen-2021-052339
Article Type:	Protocol
Date Submitted by the Author:	13-Apr-2021

Complete List of Authors:	Priebe, Stefan; Queen Mary University of London, Unit of Social and Community Psychiatry<br>Fung, Catherine ; Queen Mary University of London, Unit for Social and Community Psychiatry<br>Brusco, Luis Ignacio; Universidad de Buenos Aires, Department of Psychiatry and Mental Health, School of Medicine<br>Carbonetti, Fernando; Universidad de Buenos Aires, Department of Psychiatry and Mental Health, School of Medicine<br>Gómez-Restrepo, Carlos; Pontificia Universidad Javeriana, Department of Clinical Epidemiology and Biostatistics; Pontificia Universidad Javeriana, Department of Psychiatry and Mental Health<br>Uribe, Miguel ; Pontificia Universidad Javeriana, Department of Psychiatry and Mental Health<br>Diez-Canseco, Francisco; Universidad Peruana Cayetano Heredia, CRONICAS Center of Excellence in Chronic Diseases<br>Smuk, Melanie; London School of Hygiene & Tropical Medicine, Medical Statistics<br>Holt, Nicola; University of the West of England, Department of Health and Social Sciences<br>Kirkbride, James; University College London, Division of Psychiatry<br>Araya, Ricardo; King's College London, Health Service and Population Research<br>Morgan, Craig; King's College London, Health Service and Population Research<br>Eldridge, Sandra; Queen Mary University of London, Pragmatic Clinical Trials Unit<br>Heritage, Paul; Queen Mary University of London<br>Bird, Victoria; Queen Mary University of London, Unit for Social and Community Psychiatry

Keywords:	Depression & mood disorders < PSYCHIATRY, MENTAL HEALTH, Anxiety disorders < PSYCHIATRY

SCHOLARONE™
Manuscripts

For peer review only - http://bmjopen.bmj.com/site/about/guidelines.xhtml

I, the Submitting Author has the right to grant and does grant on behalf of all authors of the Work (as defined in the below author licence), an exclusive licence and/or a non-exclusive licence for contributions from authors who are: i) UK Crown employees; ii) where BMJ has agreed a CC-BY licence shall apply, and/or iii) in accordance with the terms applicable for US Federal Government officers or employees acting as part of their official duties; on a worldwide, perpetual, irrevocable, royalty-free basis to BMJ Publishing Group Ltd ("BMJ") its licensees and where the relevant Journal is co-owned by BMJ to the co-owners of the Journal, to publish the Work in this journal and any other BMJ products and to exploit all rights, as set out in our licence.

The Submitting Author accepts and understands that any supply made under these terms is made by BMJ to the Submitting Author unless you are acting as an employee on behalf of your employer or a postgraduate student of an affiliated institution which is paying any applicable article publishing charge ("APC") for Open Access articles. Where the Submitting Author wishes to make the Work available on an Open Access basis (and intends to pay the relevant APC), the terms of reuse of such Open Access shall be governed by a Creative Commons licence – details of these licences and which Creative Commons licence will apply to this Work are set out in our licence referred to above.

Other than as permitted in any relevant BMJ Author's Self Archiving Policies, I confirm this Work has not been accepted for publication elsewhere, is not being considered for publication elsewhere and does not duplicate material already published. I confirm all authors consent to publication of this Work and authorise the granting of this licence.

For peer review only

Which resources help young people to prevent and overcome mental distress? A programme of observational studies in deprived urban areas in Latin America

Stefan Priebe[1], Catherine Fung[1*], Luis Ignacio Brusco[2], Fernando Carbonetti[2], Carlos Gómez-Restrepo[3,4,5], Miguel Uribe[4], Francisco Diez-Canseco[6], Melanie Smuk[7], Nicola Holt[8], James B. Kirkbride[9], Ricardo Araya[10], Craig Morgan[10], Sandra Eldridge[11], Paul Heritage[12], Victoria Bird[1].

[1] Unit for Social and Community Psychiatry (WHO Collaborating Centre for Mental Health Service Development), Queen Mary University of London, United Kingdom.

[2] Department of Psychiatry and Mental Health, School of Medicine, Universidad Buenos Aires, Argentina.

[3] Department of Clinical Epidemiology and Biostatistics, Pontificia Universidad Javeriana, Bogotá, Colombia.

[4] Department of Psychiatry and Mental Health, Pontificia Universidad Javeriana, Bogotá, Colombia.

[5] Hospital Universitario San Ignacio, Bogotá, Colombia.

[6] CRONICAS Center of Excellence in Chronic Diseases, Universidad Peruana Cayetano Heredia, Lima, Peru.

[7] Department of Medical Statistics, Faculty of Epidemiology and Population Health, London School of Hygiene and Tropical Medicine, United Kingdom.

[8] Department of Health and Social Sciences, University of the West of England, Bristol, United Kingdom.

[9] Division of Psychiatry, University College London, United Kingdom.

[10] Health Service and Population Research, King's College London, United Kingdom.

For peer review only

For peer review only - http://bmjopen.bmj.com/site/about/guidelines.xhtml

[11] Pragmatic Clinical Trials Unit, Queen Mary University of London, United Kingdom.

[12] School of English and Drama, Queen Mary University of London, United Kingdom.

*Corresponding author:

Catherine Fung

Unit for Social & Community Psychiatry

WHO Collaborating Centre for Mental Health Services Development

Queen Mary University of London

E-mail address: c.fung@qmul.ac.uk

Postal address: Newham Centre for Mental Health, E13 8SP, London, United Kingdom

Word count: 3984

For peer review only - http://bmjopen.bmj.com/site/about/guidelines.xhtml

ABSTRACT

Introduction

Improving the mental health of young people is a global public health priority. In Latin America, young people living in deprived urban areas face various risk factors for mental distress. However, most either do not develop mental distress in the form of depression and anxiety, or recover within a year without treatment from mental health services. This research programme seeks to identify the personal and social resources that help young people to prevent and recover from mental distress.

Methods and analysis

A cross-sectional study will compare personal and social resources used by 1,020 young people (aged 15-16 and 20-24 years) with symptoms of depression and/or anxiety and 1,020 without. A longitudinal cohort study will follow-up those young people with mental distress after six months and after one year and compare resource-use in those who do and do not recover. An Experience Sampling Method study will intensively assess activities, experiences and mental distress in subgroups over short periods of time. Finally, case studies will be developed to highlight existing initiatives that effectively support young people to prevent and recover from mental distress. In the analysis, we will assess differences between young people with and without distress at baseline using t-tests and chi-squared tests. Within the groups with mental distress, multivariate logistic regression analyses using a random effects model will assess the relationship between predictor variables and recovery.

Ethics and dissemination

Ethics approvals are received from the ethics committees of each partner institution and Queen Mary University of London. Dissemination will include arts-based methods and target different audiences such as national stakeholders, researchers from different disciplines and the general public.

Trial registration: ISRCTN72241383 (Date of Registration: 16/12/2020).

For peer review only

Key words: global mental health, depression, anxiety, mental distress, youth, resilience, resources, longitudinal cohort, cross-sectional, experience sampling method

ARTICLE SUMMARY

Strengths and limitations of the study

- The large sample size will allow both comparisons of young people with and without distress and the exploration of factors predicting recovery in two relevant age groups.
- The inclusion of three diverse Latin American countries provides the possibility to explore commonalities and differences across different contexts.
- Inclusion of experience sampling methodology (ESM) enables exploration of resource-use in short-term recovery (within hours/days) and comparison with long-term recovery (over one year).
- Traditional methods of collecting data, i.e. completing questionnaires and in-depth interviews, may not be the most effective ways of engaging young people in research.
- The ongoing pandemic requires flexibility for online and offline data collection, which may increase the variance of findings and reduce the statistical power to identify differences between groups and predictor variables for outcomes.

INTRODUCTION

Adolescence and young adulthood are critical periods of social, behavioural and psychological maturation and change. These periods create opportunities for the development of life-long wellbeing and resilience in the face of future adversities. However, they are also associated with a significant risk for developing mental ill-health, and estimates suggest that 75% of all mental disorders begin by the age of 24[1]. The World Health Organisation (WHO) has identified improving mental health among

For peer review only - http://bmjopen.bmj.com/site/about/guidelines.xhtml

young people as a key priority required to promote sustained economic and social development[2]. Poor mental health in the form of depression and anxiety is associated with high levels of distress and disability, future physical and psychiatric morbidity and educational and social impairment[3]. Furthermore, over half of all adolescent suicides are attributable to depression[4], making it the leading cause of mortality for this age group[5].

Urban regions, predominantly large cities, account for 81% of the population in Latin America, making it one of the most urbanised regions in the world[6]. Within these environments, people are frequently exposed to various risk factors for poor mental health. These include poverty, social fragmentation, poor education, poor housing, low employment rates, gang warfare, victimisation, violence, and widespread substance misuse[7–10].

Depression and anxiety during adolescence and youth are a particular concern within low- and middle-income countries (LMICs), where the burden of common mental disorders is greatest[2,11,12]. This includes Latin America, where young people represent one-quarter of the population. Estimated levels of depression and/or anxiety for adolescents within the region range from 17% in Colombia[13] to 26% in Argentina[14]. Those exposed to adversities such as violent conflicts, internal displacement, and poverty are at an even greater risk[15]. However, despite all the risk factors, the majority of young people do not suffer from depression and/or anxiety.

Due to the scarcity of financial and human resources[16], young people in deprived areas in Latin America rarely receive formal treatment when experiencing mental distress. Yet, evidence suggests that 50-60% experience symptomatic recovery within one year[17,18]. This raises the question as to which resources individuals in these contexts mobilise to prevent and overcome mental distress and build mental health resilience.

For peer review only

For peer review only - http://bmjopen.bmj.com/site/about/guidelines.xhtml

Resilience has been defined as withstanding or overcoming adversity, trauma and stress and rebounding back from distress[19]. Thus, it covers the two processes: preventing mental distress in the face of adversity and recovering from distress if and when it develops. A systematic review into the concept of resilience within mental health classified its characteristics into two components - personal and social resources[20]. Resources encompass a wide range of strengths, assets, materials, sources of information or help and means of support available to the individual. They are commonly mobilised through and in activities. These activities may be individual and reflect the health behaviours or the skills and abilities of the person[21] (personal resources); or group-based and at the community or societal level, such as captured in the concepts of social capital, social connectedness, social identity or social networks (social resources)[22]. Resources may be specific to culture, context and locality (e.g. local music group).

Previous research has focused mainly on risk factors for developing mental disorders. However, complete primary prevention of mental distress among young people, particularly in adverse urban environments, is unrealistic. This programme therefore aims to identify which personal and social resources young people use and which ones help them to overcome mental distress, reflecting the concepts of secondary and tertiary prevention[23] as well as recovery. This crucial step may lead to future interventions to reduce the burden of mental distress in young people living in deprived urban neighbourhoods in Latin America and other LMICs and help them to maintain good mental health throughout the rest of their lives.

Programme collaborators

This research programme is coordinated by the Unit for Social and Community Psychiatry (WHO Collaborating Centre for Mental Health Service Development) at Queen Mary University of London, and conducted in collaboration with researchers from Universidad de Buenos Aires, Pontificia Universidad Javeriana (Bogotá), Universidad Peruana Cayetano Heredia (Lima), University College

For peer review only - http://bmjopen.bmj.com/site/about/guidelines.xhtml

London and King's College London. The research activities will take place in deprived areas of three large Latin American cities: Buenos Aires (Argentina), Bogotá (Colombia) and Lima (Peru).

A unique aspect of this programme is the partnership with well-established community-based arts organisations in each city: Fundación Crear Vale la Pena (Buenos Aires), Fundación Batuta and Familia Ayara (Bogotá), and Teatro La Plaza (Lima). Through specific art forms and creativity, these organisations work to support adolescents and young people living in vulnerable neighbourhoods to improve their quality of life, strengthen socio-emotional skills, and express and reflect on social realities and problems affecting young people. Arts-based activities are well-documented in their power to engage young people in research and to explore sensitive topics such as mental health, facilitate wider discussions and generate new knowledge[24,25]. The purpose of these collaborations is therefore to work through the arts to involve young people in this programme and its discourse about how to prevent and overcome mental distress; to explore the use of arts-based methodologies to understand resource-use in young people; and to use arts practices to disseminate the findings to a wider, non-academic audience.

Objectives

The overall aim is to identify which resources help young people living in deprived urban environments in Latin America prevent and recover from depression and/or anxiety.

The aim will be addressed via specific objectives:

1. Determine whether resource-use differs between adolescents and young adults with and without depression and/or anxiety,
2. Identify which resources help adolescents and young adults recover from depression and/or anxiety over a one-year period,

For peer review only

3. Explore existing approaches and resource-oriented interventions that are effective in preventing depression and/or anxiety in adolescents and young adults or in supporting them to recover.

METHODS AND ANALYSIS

Study design

The programme consists of several observational studies, using quantitative and qualitative methods (see Figure 1 for an overview of the study design). The methods have been finalised in a preparatory phase with workshops, focus groups and pilot interviews with young people in each of the three cities. The research activities are planned with procedures in place for both online and face-to-face data collection depending on the current local situation of the COVID-19 pandemic and government restrictions.

Figure 1: Study design

Settings

This study will take place in Buenos Aires (Argentina), Bogotá (Colombia) and Lima (Peru). In each city, we will select participants living in the 50% poorest neighbourhoods, defined according to comparable measures of poverty available locally: The Human Development Index[26] in Lima and Bogotá and unsatisfied basic needs[27] in Buenos Aires. Within these communities, recruitment of the younger age group will be mostly from schools and of the older group mainly from primary care centres. Other recruitment centres might include local arts centres, youth organisations, and other community and educational organisations within defined geographical areas.

Participants

For peer review only - http://bmjopen.bmj.com/site/about/guidelines.xhtml

Study participants predominantly fall into two age groups: adolescents aged 15-16 years old and young adults aged 20-24 years old, with and without mental distress, living in the defined geographical areas (see above), with capacity to provide informed consent or assent alongside parental informed consent. Exclusion criteria are any severe mental illness (psychosis, bipolar disorder, schizophrenia), cognitive impairment and illiteracy (where participants are required to self-administer questionnaires).

Cross-sectional and longitudinal cohort studies

At each of the three sites, we will recruit 340 young people (15-16 year olds and 20-24 year olds) with mental distress and 340 young people without. Using a dimensional approach – rather than diagnostic categories – mental distress will be defined by a score of greater than nine on either the Patient Health Questionnaire-8 (PHQ-8)[28] or General Anxiety Disorder-7 (GAD-7)[29]. Both scales are self-rated. The PHQ-8 assesses symptoms of depression and the GAD-7 of anxiety. Each participant will be asked to complete the developed assessment battery.

Young people identified with mental distress will be invited to take part in the longitudinal study, which will allow comparison of resource-use in those who do and do not recover. Participants will be asked to complete a brief assessment at 6 months and the same full assessment battery at 12 months (Supplementary File 1 shows the schedule of assessments).

Experience Sampling Method study

ESM is a method of data collection which asks participants to report on their thoughts, feelings, behaviours and environment on multiple occasions as they go about their daily activities each day for a specific time period, e.g. over seven days[30]. ESM allows for a detailed assessment of the interaction between real-world context and phenomena that is unaffected by issues of recall[30]. Data will be

For peer review only - http://bmjopen.bmj.com/site/about/guidelines.xhtml

collected using a mobile phone app called eMoodie[31], which was developed specifically for young people. The ESM study will run alongside the cross-sectional and longitudinal studies sharing the same aims and with the additional aim to determine whether young people use similar personal and social resources for short-term recovery (within hours or days) as for long-term recovery (over one year).

The ESM study will recruit 150 participants (50 in each country) already enrolled in the cross-sectional and longitudinal studies: 90 with depression and/or anxiety and 60 without, as defined above and across the two age groups (15-16 year olds and 20-24 year olds). Participants will first attend a training session with a researcher, who will explain how to use the eMoodie app, when to complete the ESM assessments, to practise the questionnaires and explain each item in detail. Participants will be given phones, and/or data cards to access the internet if this is needed. Participants can contact the researcher with any questions about, or problems with, eMoodie or the ESM questions. The researcher will conduct a midweek phone call at an agreed time to discuss any concerns or problems they are experiencing with completing the questionnaires and motivate participants to complete as many ESM questionnaires as possible.

Young adult participants (20-24 years old) will complete the ESM assessment 8 times per day over 7 days, and adolescent participants (15-16 years old) will complete the ESM assessment 5 times per day for school days and 8 times per day for weekend days, again over 7 days. The times will be scheduled at random within set blocks of time. At the end of the 7-day data collection period, participants will be asked to answer a short questionnaire to briefly explore through open questions, their experience of the ESM study and any problems encountered.

The 90 participants defined with depression and/or anxiety, who participated in both the ESM study baseline and longitudinal study, will be contacted to complete the ESM assessments again after 12 months.

Case studies of good practice

For peer review only - http://bmjopen.bmj.com/site/about/guidelines.xhtml

This work activity will support knowledge exchange and suggest practical implications. Case studies of good practice models will illustrate and explain our previous findings. Based on the longitudinal study, we will identify and describe small-scale interventions already happening within the communities (e.g. adolescent wellbeing centres), and health and education services (e.g. school-based resilience programmes), in order to assess how communities and services can effectively prevent or respond to mental distress in young people.

Arts-based laboratories will run across 6-8 weeks by the partner arts organisations in each city to explore attitudes and perceptions about mental distress in 30 participants from the longitudinal study (10 in each city) in order to reveal individual, social and contextual resilience factors and the significance of different personal and social resources in their own recovery from depression and/or anxiety. The methodologies developed in the preparatory work for researcher participation/observation will be applied at key moments throughout the workshop programme and in the discussions with young people and the arts facilitators from each organisation. Longitudinal study participants will also have the opportunity to participate in integrated arts workshops with other young people in each arts organisation.

To identify areas of good practice, 30 longitudinal study participants will be invited to take part in in-depth interviews as soon as possible after completing the 12 month follow-up. Interviews will explore which resources individuals used and found helpful or not helpful, which resources they are aware of, and any suggestions for ways in which the community or/and services could help them with their recovery. These interviews will be used alongside a stakeholder consultation workshop to recommend areas of good practice within each city, including different types of services, initiatives, projects or approaches. This workshop will be conducted with facilitated discussion asking for visual and written descriptions of existing projects and approaches. Field visits and interviews with individuals who work at and/or use these initiatives will be conducted to aid scale-up and dissemination. Finally, a

For peer review only - http://bmjopen.bmj.com/site/about/guidelines.xhtml

stakeholder consultation will bring together the information collected to produce the case studies of each approach.

Measures

For the cross-sectional and longitudinal studies, data will be collected using a case report form (CRF). Each country-specific CRF contains the relevant language variations. A small number of questions will be varied to collect locally relevant data (socio-demographic characteristics, health insurances) and in-line with local regulations (sexual activities). All countries will be assessing the same key outcomes and resources: personal background and characteristics, social context, mental distress, quality of life, life events, experiences and the actual use of personal resources and social resources. Supplementary File 1 summarises the questionnaires and the data collected at baseline, 6- and 12-months.

At baseline, sociodemographic data will be collected, including information about age, gender, living situation, current or obtained level of education, employment, current and previous experiences of depression and anxiety, and family history of depression and anxiety. Variables that might change over a one-year period will be collected again at the 12 month follow-up, including current main occupation and current experiences of depression and anxiety.

The ESM assessments will ask participants about their current affect, arousal state, location, company, and activity (see Supplementary File 2).

Bias

Adolescents will be primarily recruited from schools and young adults from primary care centres to ensure we recruit a varied sample of young people. Samples for both age groups will include a balance of gender. Resource-use may be affected by the presence of the pandemic and restrictions to leaving

For peer review only - http://bmjopen.bmj.com/site/about/guidelines.xhtml

For peer review only

homes during lockdown, but we will assess resource-use as planned and collect information about lockdown in each city throughout data collection, to facilitate further data analysis as required.

Sample size calculations

The sample size for the cross-sectional and longitudinal studies has been calculated for identifying variables that predict recovery over a one-year period. Assuming a predictor variable (personal or social resource) exists in 10% of the population, we can detect a difference in recovery rate between 40% and 60% with 90% power and at a 5% significance level with a total sample of 762 people. Assuming a dropout rate of 25%, 1016 participants with mental distress are required at baseline. Recruiting 340 people with mental distress in each of the three countries provides a total sample of 1020.

Patient and public involvement

The study is not a clinical study recruiting patients. Participants will be young people with and without mental distress in the deprived communities in Latin American cities. Young people in Bogotá, Buenos Aires and Lima were extensively involved in the preparation of the study. In arts-based workshops, focus groups and pilots of the assessment battery, they helped to develop appropriate methods for assessing resource-use. For the arts-based workshops and the arts laboratories, we are working with our partner arts organisations, which use a variety of artistic languages that can encourage engagement in research. Focus groups with adolescents (aged 15-16 years), young adults (aged 20-24 years), and professionals (mental health professionals, educators, staff from youth organisations) explored views on resources that young people use to prevent and overcome mental distress. Pilots of the assessment battery established the feasibility and acceptability of an assessment battery, including the burden of completing this through timed pilots. The findings from the preparatory work

For peer review only - http://bmjopen.bmj.com/site/about/guidelines.xhtml

allowed us to refine the assessment measures to reflect young people's experiences, priorities and preferences. The overall research question and design of the study were informed by the literature and expertise of the multi-disciplinary research team, and patients/the public were not involved in this process.

Each research team has established a Lived Experience Advisory Panel (LEAP) comprised of young people aged 15-24 years old with lived experience of depression and/or anxiety. The purpose of the LEAPs is to engage young people and ensure their voice is present and heard throughout the research, including recruitment to and conduct of the research. The LEAP panel and our partner arts organisations will be consulted about the dissemination of findings to wider audiences of young people to continue the discourse about mental health distress.

Data analysis

Local researchers will transcribe verbatim the audio or video recordings from the in-depth interviews and will analyse using Framework Analysis following the stages of familiarisation; identifying a thematic framework; indexing; charting; and interpretation[32]. This will focus on common features of the identified practices and resources, which could be scaled up and more widely disseminated.

For the cross-sectional and longitudinal studies, data will be collected on paper CRFs with pseudonymised data entered onto a secure online database called REDCap, and/or participants will complete the electronic form that captures data directly onto the same database. Descriptive statistics will be reported for socio-demographic data for all participants. The numbers of individuals at each stage of the study will be reported, including the numbers potentially eligible, screened, confirmed as eligible, included, followed-up and analysed.

For the cross-sectional study, differences between the groups on each of the measures will be assessed using t-tests and chi-squared tests to determine whether resource-use differs between

For peer review only - http://bmjopen.bmj.com/site/about/guidelines.xhtml

young people with and without depression and/or anxiety and whether the differences are apparent for the two age groups and gender.

We understand long-term recovery as recovery over a one-year period. At one-year follow up, based on the PHQ-8 and GAD-7 scores, individuals will be classified as recovered – defined as no longer screening positive for depression and/or anxiety (scoring 9 or less on either scale), or not recovered (scoring greater than 9 on either scale). The distribution and level of resources will be summarised using the median, whilst frequency and counts will summarise exposure to different community resources. A multivariate logistic regression analysis using a random effects model will assess the relationship between predictor variables and recovery, firstly for each age group, and then including the whole sample to determine whether the same resources are associated with recovery for all age groups and genders.

Data from the ESM assessments will be collected using the eMoodie mobile phone app, and then stored on a secure server. The data will be multilevel as multiple observations are nested within participants. Short-term recovery means any recovery within the 7-day assessment period, whether it is within hours or over the full seven days. Recovery will be assessed as a continuous variable, as improvement of any reported distress. The analysis of this will be exploratory and consider different levels of distress and improvement. Long-term recovery will use the same definition as described above. Multilevel models will assess the link between stress-related variables and negative and positive affect, controlling for potential confounding factors such as gender and sociodemographic variables. Differences between groups will be assessed, and different types of resources will be categorised and the frequency of resources within each category calculated.

ETHICS AND DISSEMINATION

For peer review only - http://bmjopen.bmj.com/site/about/guidelines.xhtml

For all research activities, informed consent will be obtained from participants, or assent will be obtained from participants under 18 years old alongside informed consent from their parent or legal guardian. Each research team will provide participants with information about depression and anxiety, and organisations and resources in each city for mental health support. Procedures are in place to manage any instances of risk of harm to participants or others. Where researchers conduct face-to-face data collection, they are required to follow their institutional and/or government guidelines to protect themselves and participants from COVID-19 infection. All data will be pseudonymised, handled and stored in line with the Data Protection Act 2018, General Data Protection Regulation, and national data protection laws in each partner country. Only research team members will have access to data during the study, which will be stored on REDCap, hosted on secure servers at Queen Mary, University of London. To protect the identity of participants, they will each be assigned a Participant Identification Number (PIN). The list linking participants' names to their PIN will be stored separately and securely. After the completion of the programme, the datasets used and/or analysed during the cross-sectional, longitudinal and ESM studies will be available in a publically available repository. Participants' consent (or their parent/guardian's consent if under 18 years old) to share de-identified will be obtained for this purpose.

The findings will be disseminated to national stakeholders, including policymakers, professionals in healthcare and education, charities and Non-Governmental Organisations. Traditional routes of dissemination include publishing open access in peer-reviewed journals and presenting findings at national and international conferences. Through our partner arts organisations, we will also enable young people to disseminate findings to a wider, non-academic audience, including their own peer groups.

Abbreviations

For peer review only - http://bmjopen.bmj.com/site/about/guidelines.xhtml

LMICs- Low- and middle-income countries; WHO- World Health Organisation; ESM- Experience Sampling Method;; LEAP- Lived Experience Advisory Panel; PHQ-8- Patient Health Questionnaire-8; GAD-7- General Anxiety Disorder-7; CRF- Case Report Form; PIN- Participant Identification Number; UK- United Kingdom.

DECLARATIONS

Ethics approval

For the cross-sectional, longitudinal and ESM studies and interviews with longitudinal study participants, written approval was received:

1. From the Ethics Committee in Biomedical Research, Alberto C. Taquini Institute for Translational Medicine Research, Faculty of Medicine, University of Buenos Aires on 10/11/2020.
2. From the Faculty of Medicine - Research and Ethics Committee of the Pontificia Universidad Javeriana, Bogota on 20/11/2020 (ref: FM-CIE-1138-20)
3. From the Institutional Ethics Committee of Research of the Universidad Peruana Cayetano Heredia on 16/11/2020 (ref: 581-33-20)
4. From the Queen Mary Ethics of Research Committee on 16/11/2020 (ref: QMERC2020/02).

The study recruited its first participant in Bogotá on 26th February 2021. Data collection is yet to begin.

Data statement

The datasets used and/or analysed during the cross-sectional, longitudinal and ESM studies will be available in a publically available repository.

Acknowledgements

For peer review only

We would like to acknowledge our partner arts organisations for their ongoing contribution to the OLA programme: Fundación Crear Vale la Pena (Buenos Aires), Fundación Batuta and Familia Ayara.

Author contributions

SP and VB led on the conception, design and organisation of the programme. CF and SP wrote the first draft of this manuscript. All authors contributed to the whole study design, commented on earlier drafts, and read and approved the manuscript. MS and SE particularly contributed to the statistical aspects of the programme, JBK to the method for identifying appropriate geographical areas, PH to the planning of all arts activities, NH to the adaptation of the Experience Sampling Method, CM to conceptual and methodological issues of the cohort study, RA to considerations of the Latin American context, and CF to organisational aspects of the programme. LIB, FC, CGR, JMUR, and FDC specifically contributed to the adaptation of the design to the national contexts in Argentina, Colombia and Peru.

Funding

This work is supported by the Medical Research Council (grant number: MR/S03580X/1)

(Bogotá), and Teatro La Plaza (Lima).

Competing interests

The authors declare that they have no competing interests

References

1. Kessler RC, Berglund P, Demler O, et al. Lifetime prevalence and age-of-onset distributions of DSM-IV disorders in the national comorbidity survey replication. *Arch Gen Psychiatry*. 2005;62(7):768. doi:10.1001/archpsyc.62.6.593

2. Varese F, Smeets F, Drukker M, et al. Childhood adversities increase the risk of psychosis: A meta-analysis of patient-control, prospective-and cross-sectional cohort studies. *Schizophr*

For peer review only - http://bmjopen.bmj.com/site/about/guidelines.xhtml

Bull. 2012;38(4). doi:10.1093/schbul/sbs050

3. Merikangas KR, He JP, Burstein M, et al. Lifetime prevalence of mental disorders in U.S. adolescents: Results from the national comorbidity study-adolescent supplement (NCS-A). *J Am Acad Child Adolesc Psychiatry*. 2010;49(10):980-989. doi:10.1016/j.jaac.2010.05.017

4. Thompson MP, Kingree JB, Lamis D. Associations of adverse childhood experiences and suicidal behaviors in adulthood in a U.S. nationally representative sample. *Child Care Health Dev*. 2019;45(1):121-128. doi:10.1111/cch.12617

5. Horn AB, Cañizares C, Gómez Y. Prevention of adolescent depression in the Spanish-speaking world. *Int J Environ Res Public Health*. 2014;11(6):5665-5683. doi:10.3390/ijerph110605665

6. United Nations, Department of Economic and Social Affairs PD. *World Urbanization Prospects 2018: Highlights*.; 2019. doi:10.18356/6255ead2-en

7. Patel V, Flisher AJ, Hetrick S, et al. Mental health of young people: a global public-health challenge. *Lancet*. 2007;369(9569):1302-1313. doi:10.1016/S0140-6736(07)60368-7

8. Kim D. Blues from the neighborhood? Neighborhood characteristics and depression. *Epidemiol Rev*. 2008;30(1). doi:10.1093/epirev/mxn009

9. Morgan C, Reininghaus U, Reichenberg A, et al. Adversity, cannabis use and psychotic experiences: Evidence of cumulative and synergistic effects. *Br J Psychiatry*. 2014;204(5). doi:10.1192/bjp.bp.113.134452

10. Thapar A, Collishaw S, Pine DS, et al. Depression in adolescence. *Lancet*. 2012;379(9820). doi:10.1016/S0140-6736(11)60871-4

11. Zammit S, Lewis G, Dalman C, et al. Examining interactions between risk factors for psychosis. *Br J Psychiatry*. 2010;197(3):207-211. doi:10.1192/bjp.bp.109.070904

12. Ferrari AJ, Charlson FJ, Norman RE, et al. Burden of Depressive Disorders by Country, Sex,

For peer review only

For peer review only - http://bmjopen.bmj.com/site/about/guidelines.xhtml

Age, and Year: Findings from the Global Burden of Disease Study 2010. *PLoS Med*. 2013;10(11). doi:10.1371/journal.pmed.1001547

13. Murcia EH, Losada YL, Rojas LA, et al. Prevalencia de la depresión infantil en neiva (Colombia). *Av en Psicol Latinoam*. 2009;27(1).

14. Méndez X, Rosa AI, Montoya M, et al. Tratamiento psicológico de la depresión infantil y adolescente: ¿Evidencia o promesa? *Psicol Conductual*. 2002;10(3).

15. Navarro L, Moscoso J, De La Cruz G. La investigación de la depresión en adolescentes en el Perú: una revisión sistemática. *Lib Rev Peru Psicol*. 2017;23(1). doi:10.24265/liberabit.2017.v23n1.04

16. Rathod S, Pinninti N, Irfan M, et al. Mental Health Service Provision in Low- and Middle-Income Countries. *Heal Serv Insights*. 2017;10:1-7. doi:10.1177/1178632917694350

17. March JS, Silva S, Petrycki S. Fluoxetine, cognitive-behavioral therapy, and their combination for adolescents with depression: Treatment for Adolescents with Depression Study (TADS) randomized controlled trial. *J Am Med Assoc*. 2004;292(7):807-820. doi:10.1001/jama.292.7.807

18. Stapinski LA, Montgomery AA, Heron J, et al. Depression Symptom Trajectories and Associated Risk Factors among Adolescents in Chile. *PLoS One*. 2013;8(10). doi:10.1371/journal.pone.0078323

19. Anderson K, Priebe S. Concepts of resilience in adolescent mental health research. *J Adolesc Heal*. in press.

20. Gross JJ, John OP. Individual Differences in Two Emotion Regulation Processes: Implications for Affect, Relationships, and Well-Being. *J Pers Soc Psychol*. 2003;85(2):348-362. doi:10.1037/0022-3514.85.2.348

For peer review only

For peer review only - http://bmjopen.bmj.com/site/about/guidelines.xhtml

21. Pargas RCM, Brennan PA, Hammen C, et al. Resilience to Maternal Depression in Young Adulthood. *Dev Psychol*. 2010;46(4):805-814. doi:10.1037/a0019817

22. Kim J. Neighborhood disadvantage and mental health: The role of neighborhood disorder and social relationships. *Soc Sci Res*. 2010;39(2):260-271. doi:10.1016/j.ssresearch.2009.08.007

23. Simeonsson RJ. Primary, Secondary, and Tertiary Prevention in Early Intervention. *J Early Interv*. 1991;15(2). doi:10.1177/105381519101500202

24. Fenge LA, Hodges C, Cutts W. Seen But Seldom Heard: Creative Participatory Methods in a Study of Youth and Risk. *Int J Qual Methods*. 2011;10(4). doi:10.1177/160940691101000408

25. Yonas MA, Burke JG, Miller E. Visual Voices: A Participatory Method for Engaging Adolescents in Research and Knowledge Transfer. *Clin Transl Sci*. 2013;6(1). doi:10.1111/cts.12028

26. United Nations Development Programme Human Development Reports. Accessed January 13, 2021. http://hdr.undp.org/en/content/human-development-index-hdi

27. Santos ME. *Measuring Multidimensional Poverty in Latin America: Previous Experience and the Way Forward*.; 2014.

28. Kroenke K, Strine TW, Spitzer RL, et al. The PHQ-8 as a measure of current depression in the general population. *J Affect Disord*. 2009;114(1-3). doi:10.1016/j.jad.2008.06.026

29. Spitzer RL, Kroenke K, Williams JBW, et al. A brief measure for assessing generalized anxiety disorder: The GAD-7. *Arch Intern Med*. 2006;166(10). doi:10.1001/archinte.166.10.1092

30. Myin-Germeys I, Kasanova Z, Vaessen T, et al. Experience sampling methodology in mental health research: new insights and technical developments. *World Psychiatry*. 2018;17(2):123-132. doi:10.1002/wps.20513

31. Domoff SE, Banga CA, Borgen AL, et al. Use of passive sensing to quantify adolescent mobile device usage: Feasibility, acceptability, and preliminary validation of the eMoodie application.

For peer review only

For peer review only - http://bmjopen.bmj.com/site/about/guidelines.xhtml

Hum Behav Emerg Technol. Published online 2021. doi:10.1002/hbe2.247

32. Richie J, Spencer L. Qualitative data analysis for applied policy research. In A. Bryman & R.G. Burgess (Eds). In: *Analyzing Qualitative Data*. ; 1994.

For peer review only

Figure 1: Study design

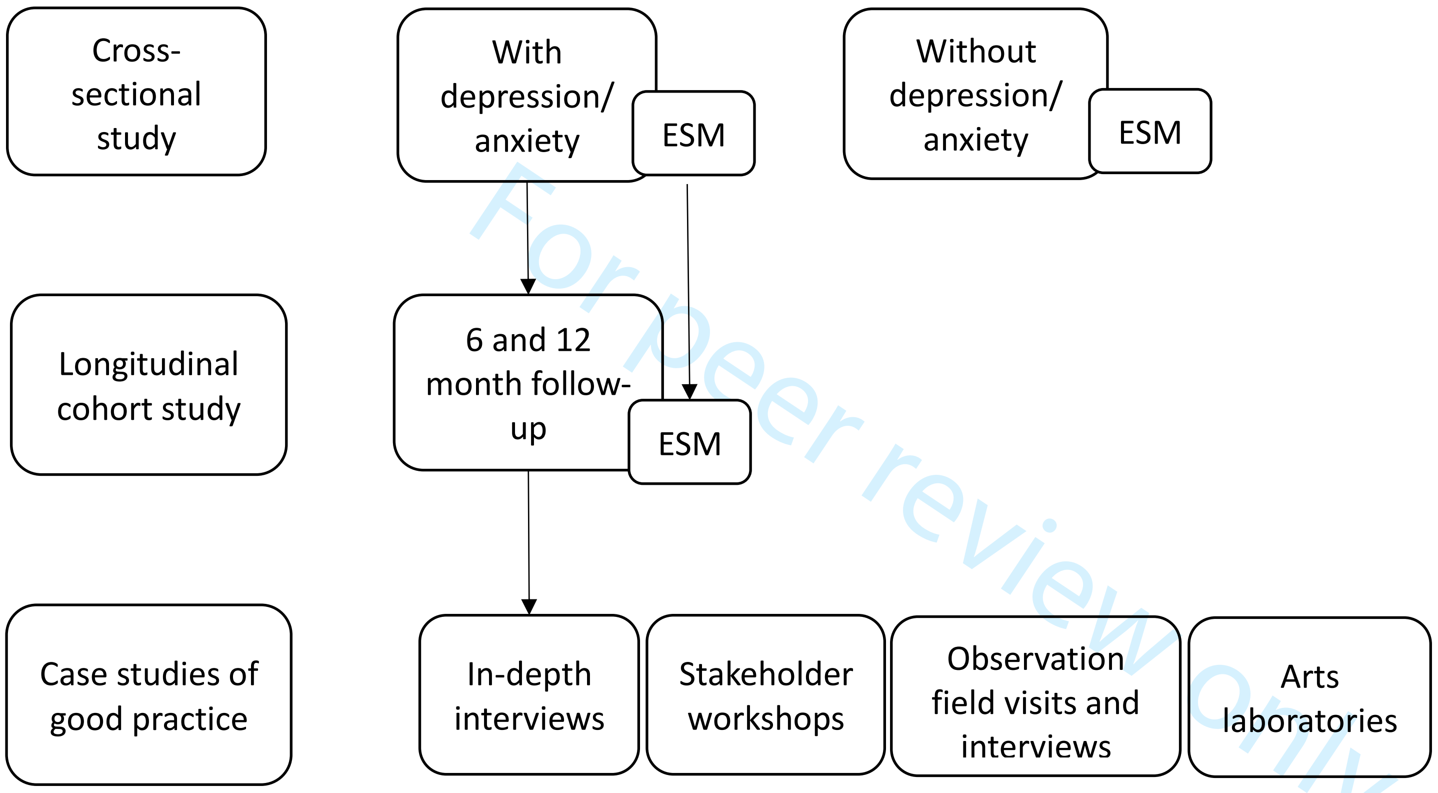

For peer review only

Supplementary File 1: Schedule of assessments for the cross-sectional and longitudinal studies

Item measured	Scale/ assessment method	Description of scale	Baseline	6-month follow-up	12-month follow-up
**DEMOGRAPHICS**					
Demographics	Current living situation	Includes variables like gender, age, education level etc	X		X*
	History of depression and anxiety	Participants' and their parents' experiences of mental distress and treatment received	X		X*
**MEASURES OF MENTAL DISTRESS**					
Degree of distress	Patient Health Questionnaire-8 (PHQ-8)	Measures degree of experiencing a list of symptoms associated with depression	X	X	X
	Generalised Anxiety Disorder-7 (GAD-7)	Measures degree of experiencing a list of symptoms associated with anxiety	X	X	X
Drug use	The Alcohol, Smoking and Substance Involvement Screening Test (ASSIST) - adapted	Measures drug use in the lifetime and last 3 months	X		X
	Adapted Teen Addiction Severity Index (T-ASI) - adapted	Measures drug use including alcohol use and severity	X		X
Life events	Adolescent appropriate life events scale - adapted	Captures experience of life events in their lifetime and in the last year or six month respectively	X	X	X
**MEASURES OF RESOURCES**					
General resources	Open question	What participants do when they feel mentally distressed	X		X
Impact of COVID-19	Closed questions	Brief assessment of how COVID-19 has impacted on various activities	X	TBC	TBC
**MEASURES OF PERSONAL RESOURCES**					
Quality of life	Manchester Short Assessment of Quality of Life (MANSA)	Measures perception of how satisfied they are with different aspects of their lives	X		X
Sex life	Closed question	Whether they have had sex with another person in the last month	X		X
Coping style	Child's Coping Strategy Checklist	Measures how individuals deal with problems and stress	X		X
Resilience	Connor-Davidson Resilience Scale (CD-RISC 10)	Measures resilience in response to stressful events, tragedy or trauma	X		X
**MEASURES OF SOCIAL RESOURCES**					
Use of healthcare and other services	Client Service Receipt Inventory (CSRI) - adapted	Measures frequency of use of healthcare and social services	X	X	X
Social support	Scale of Perceived Social Support	Measures perception of social support network	X		X

Social capital	Adapted Social Capital Assessment Tool (ASCAT) - adapted	Asks about perceptions of and engagement with community groups	X		X
Family support	Open questions	Asks which family member they speak to about feelings and emotions, and which family members speak to them about feelings and emotions	X		X
**MEASURES OF ACTIVITIES**					
Sports activity	Open/closed questions	Asks about sports activities including frequency of participation and the nature of these activities	X		X
Arts activity	Open/closed questions	Asks about arts activities including frequency of participation and the nature of these activities	X		X
Internet use	Question 59 from REACH study - adapted	Measures internet use via agreement with a list of statements	X		X

Legend:
X: questionnaire included
X: various items included (e.g. only sociodemographic items that might have changed will be included)*
TBC: to be confirmed depending on the COVID-19 situation in partner countries

For peer review only

Supplementary File 2: Summary of questions that form the ESM assessment

Item measured	Question	Description
Location	Where are you now?	1 item asking participants to state their location. Participants choose from predefined categories or select "other" and provide more detail
Activity	What are you doing now?<br>Time spent doing this activity since last "beep" (notification)?	1 open question asking participants what they are doing (main activity)<br>1 item asking the participants to indicate time spent doing this activity since the last notification
Company	Who are you doing this activity with?	1 item asking participants to state who they are doing their main activity with. Participants choose from predefined categories or select "other" and provide more detail.
Affect	How are you feeling now?	1 item asks participants to rate how they are feeling on a 7-point Likert scale ranging from 1 ("extremely unhappy") to 7 ("extremely happy")
Arousal	How nervous or relaxed are you now?	1 item asking participants to rate how nervous/relaxed they are feeling on a 7-point Likert scale ranging from 1 "very nervous" to 7 "very relaxed"

For peer review only

STROBE 2007 (v4) checklist of items to be included in reports of observational studies in epidemiology*
Checklist for cohort, case-control, and cross-sectional studies (combined)

Section/Topic	Item #	Recommendation	Reported on page #
**Title and abstract**	1	(*a*) Indicate the study's design with a commonly used term in the title or the abstract	2
		(*b*) Provide in the abstract an informative and balanced summary of what was done and what was found	2,3
**Introduction**			
Background/rationale	2	Explain the scientific background and rationale for the investigation being reported	4,5
Objectives	3	State specific objectives, including any pre-specified hypotheses	6,7
**Methods**			
Study design	4	Present key elements of study design early in the paper	7
Setting	5	Describe the setting, locations, and relevant dates, including periods of recruitment, exposure, follow-up, and data collection	7-10
Participants	6	(*a*) *Cohort study*—Give the eligibility criteria, and the sources and methods of selection of participants. Describe methods of follow-up<br>*Case-control study*—Give the eligibility criteria, and the sources and methods of case ascertainment and control selection. Give the rationale for the choice of cases and controls<br>*Cross-sectional study*—Give the eligibility criteria, and the sources and methods of selection of participants	8
		(*b*) *Cohort study*—For matched studies, give matching criteria and number of exposed and unexposed<br>*Case-control study*—For matched studies, give matching criteria and the number of controls per case	
Variables	7	Clearly define all outcomes, exposures, predictors, potential confounders, and effect modifiers. Give diagnostic criteria, if applicable	11
Data sources/ measurement	8*	For each variable of interest, give sources of data and details of methods of assessment (measurement). Describe comparability of assessment methods if there is more than one group	Additional File 2
Bias	9	Describe any efforts to address potential sources of bias	12
Study size	10	Explain how the study size was arrived at	11, 12
Quantitative variables	11	Explain how quantitative variables were handled in the analyses. If applicable, describe which groupings were chosen and why	14
Statistical methods	12	(*a*) Describe all statistical methods, including those used to control for confounding	13, 14
		(*b*) Describe any methods used to examine subgroups and interactions	14
		(*c*) Explain how missing data were addressed	
		(*d*) *Cohort study*—If applicable, explain how loss to follow-up was addressed<br>*Case-control study*—If applicable, explain how matching of cases and controls was addressed	

For peer review only

		Cross-sectional study—If applicable, describe analytical methods taking account of sampling strategy	
		(*e*) Describe any sensitivity analyses	
**Results**			Section is N/A
Participants	13*	(a) Report numbers of individuals at each stage of study—eg numbers potentially eligible, examined for eligibility, confirmed eligible, included in the study, completing follow-up, and analysed	
		(b) Give reasons for non-participation at each stage	
		(c) Consider use of a flow diagram	
Descriptive data	14*	(a) Give characteristics of study participants (eg demographic, clinical, social) and information on exposures and potential confounders	
		(b) Indicate number of participants with missing data for each variable of interest	
		(c) *Cohort study*—Summarise follow-up time (eg, average and total amount)	
Outcome data	15*	*Cohort study*—Report numbers of outcome events or summary measures over time	
		Case-control study—Report numbers in each exposure category, or summary measures of exposure	
		Cross-sectional study—Report numbers of outcome events or summary measures	
Main results	16	(*a*) Give unadjusted estimates and, if applicable, confounder-adjusted estimates and their precision (eg, 95% confidence interval). Make clear which confounders were adjusted for and why they were included	
		(*b*) Report category boundaries when continuous variables were categorized	
		(*c*) If relevant, consider translating estimates of relative risk into absolute risk for a meaningful time period	
Other analyses	17	Report other analyses done—eg analyses of subgroups and interactions, and sensitivity analyses	
**Discussion**			Section is N/A
Key results	18	Summarise key results with reference to study objectives	
Limitations	19	Discuss limitations of the study, taking into account sources of potential bias or imprecision. Discuss both direction and magnitude of any potential bias	
Interpretation	20	Give a cautious overall interpretation of results considering objectives, limitations, multiplicity of analyses, results from similar studies, and other relevant evidence	
Generalisability	21	Discuss the generalisability (external validity) of the study results	
**Other information**			
Funding	22	Give the source of funding and the role of the funders for the present study and, if applicable, for the original study on which the present article is based	18

*Give information separately for cases and controls in case-control studies and, if applicable, for exposed and unexposed groups in cohort and cross-sectional studies.
Note: An Explanation and Elaboration article discusses each checklist item and gives methodological background and published examples of transparent reporting. The STROBE checklist is best used in conjunction with this article (freely available on the Web sites of PLoS Medicine at http://www.plosmedicine.org/, Annals of Internal Medicine at http://www.annals.org/, and Epidemiology at http://www.epidem.com/). Information on the STROBE Initiative is available at www.strobe-statement.org.

For peer review only

BMJ Open

Which resources help young people to prevent and overcome mental distress in deprived urban areas in Latin America? A protocol for a prospective cohort study

Journal:	*BMJ Open*
Manuscript ID	bmjopen-2021-052339.R1
Article Type:	Protocol
Date Submitted by the Author:	07-Aug-2021

Complete List of Authors:	Priebe, Stefan; Queen Mary University of London, Unit of Social and Community Psychiatry<br>Fung, Catherine ; Queen Mary University of London, Unit for Social and Community Psychiatry<br>Brusco, Luis Ignacio; Universidad de Buenos Aires, Department of Psychiatry and Mental Health, School of Medicine<br>Carbonetti, Fernando; Universidad de Buenos Aires, Department of Psychiatry and Mental Health, School of Medicine<br>Gómez-Restrepo, Carlos; Pontificia Universidad Javeriana, Department of Clinical Epidemiology and Biostatistics; Pontificia Universidad Javeriana, Department of Psychiatry and Mental Health<br>Uribe, Miguel ; Pontificia Universidad Javeriana, Department of Psychiatry and Mental Health<br>Diez-Canseco, Francisco; Universidad Peruana Cayetano Heredia, CRONICAS Center of Excellence in Chronic Diseases<br>Smuk, Melanie; London School of Hygiene & Tropical Medicine, Medical Statistics<br>Holt, Nicola; University of the West of England, Department of Health and Social Sciences<br>Kirkbride, James; University College London, Division of Psychiatry<br>Araya, Ricardo; King's College London, Health Service and Population Research<br>Morgan, Craig; King's College London, Health Service and Population Research<br>Eldridge, Sandra; Queen Mary University of London, Pragmatic Clinical Trials Unit<br>Heritage, Paul; Queen Mary University of London<br>Bird, Victoria; Queen Mary University of London, Unit for Social and Community Psychiatry

**Primary Subject Heading**:	Mental health
Secondary Subject Heading:	Global health
Keywords:	Depression & mood disorders < PSYCHIATRY, Anxiety disorders < PSYCHIATRY, MENTAL HEALTH

I, the Submitting Author has the right to grant and does grant on behalf of all authors of the Work (as defined in the below author licence), an exclusive licence and/or a non-exclusive licence for contributions from authors who are: i) UK Crown employees; ii) where BMJ has agreed a CC-BY licence shall apply, and/or iii) in accordance with the terms applicable for US Federal Government officers or employees acting as part of their official duties; on a worldwide, perpetual, irrevocable, royalty-free basis to BMJ Publishing Group Ltd ("BMJ") its licensees and where the relevant Journal is co-owned by BMJ to the co-owners of the Journal, to publish the Work in this journal and any other BMJ products and to exploit all rights, as set out in our licence.

The Submitting Author accepts and understands that any supply made under these terms is made by BMJ to the Submitting Author unless you are acting as an employee on behalf of your employer or a postgraduate student of an affiliated institution which is paying any applicable article publishing charge ("APC") for Open Access articles. Where the Submitting Author wishes to make the Work available on an Open Access basis (and intends to pay the relevant APC), the terms of reuse of such Open Access shall be governed by a Creative Commons licence – details of these licences and which Creative Commons licence will apply to this Work are set out in our licence referred to above.

Other than as permitted in any relevant BMJ Author's Self Archiving Policies, I confirm this Work has not been accepted for publication elsewhere, is not being considered for publication elsewhere and does not duplicate material already published. I confirm all authors consent to publication of this Work and authorise the granting of this licence.

For peer review only

Which resources help young people to prevent and overcome mental distress in deprived urban areas in Latin America? A protocol for a prospective cohort study

Stefan Priebe[1], Catherine Fung[1*], Luis Ignacio Brusco[2], Fernando Carbonetti[2], Carlos Gómez-Restrepo[3,4,5], Miguel Uribe[4], Francisco Diez-Canseco[6], Melanie Smuk[7], Nicola Holt[8], James B. Kirkbride[9], Ricardo Araya[10], Craig Morgan[10], Sandra Eldridge[11], Paul Heritage[12], Victoria Bird[1].

[1] Unit for Social and Community Psychiatry (WHO Collaborating Centre for Mental Health Service Development), Queen Mary University of London, United Kingdom.

[2] Department of Psychiatry and Mental Health, School of Medicine, Universidad Buenos Aires, Argentina.

[3] Department of Clinical Epidemiology and Biostatistics, Pontificia Universidad Javeriana, Bogotá, Colombia.

[4] Department of Psychiatry and Mental Health, Pontificia Universidad Javeriana, Bogotá, Colombia.

[5] Hospital Universitario San Ignacio, Bogotá, Colombia.

[6] CRONICAS Center of Excellence in Chronic Diseases, Universidad Peruana Cayetano Heredia, Lima, Peru.

[7] Department of Medical Statistics, Faculty of Epidemiology and Population Health, London School of Hygiene and Tropical Medicine, United Kingdom.

[8] Department of Health and Social Sciences, University of the West of England, Bristol, United Kingdom.

[9] Division of Psychiatry, University College London, United Kingdom.

[10] Health Service and Population Research, King's College London, United Kingdom.

For peer review only

For peer review only - http://bmjopen.bmj.com/site/about/guidelines.xhtml

[11] Pragmatic Clinical Trials Unit, Queen Mary University of London, United Kingdom.

[12] School of English and Drama, Queen Mary University of London, United Kingdom.

*Corresponding author:

Catherine Fung

Unit for Social & Community Psychiatry

WHO Collaborating Centre for Mental Health Services Development

Queen Mary University of London

E-mail address: c.fung@qmul.ac.uk

Postal address: Newham Centre for Mental Health, E13 8SP, London, United Kingdom

Word count: 3999

For peer review only

ABSTRACT

Introduction

Improving the mental health of young people is a global public health priority. In Latin America, young people living in deprived urban areas face various risk factors for mental distress. However, most either do not develop mental distress in the form of depression and anxiety, or recover within a year without treatment from mental health services. This research programme seeks to identify the personal and social resources that help young people to prevent and recover from mental distress.

Methods and analysis

A cross-sectional study will compare personal and social resources used by 1,020 young people (aged 15-16 and 20-24 years) with symptoms of depression and/or anxiety and 1,020 without. A longitudinal cohort study will follow-up young people with mental distress after six months and one year and compare resource-use in those who do and do not recover. An Experience Sampling Method study will intensively assess activities, experiences and mental distress in subgroups over short time periods. Finally, we will develop case studies highlighting existing initiatives that effectively support young people to prevent and recover from mental distress. The analysis will assess differences between young people with and without distress at baseline using t-tests and chi-squared tests. Within the groups with mental distress, multivariate logistic regression analyses using a random effects model will assess the relationship between predictor variables and recovery.

Ethics and dissemination

Ethics approvals are received from Ethics Committee in Biomedical Research, Faculty of Medicine, University of Buenos Aires; Faculty of Medicine - Research and Ethics Committee of the Pontificia Universidad Javeriana, Bogotá; Institutional Ethics Committee of Research of the Universidad Peruana Cayetano Heredia; and Queen Mary Ethics of Research Committee. Dissemination will include arts-

For peer review only

based methods and target different audiences such as national stakeholders, researchers from different disciplines and the general public.

Trial registration: ISRCTN72241383 (Registration date: 16/12/2020).

Key words: global mental health, depression, anxiety, mental distress, youth, resilience, resources, longitudinal cohort, cross-sectional, experience sampling method

ARTICLE SUMMARY

Strengths and limitations of the study

- The large sample size will allow both comparisons of young people with and without distress and the exploration of factors predicting recovery in two relevant age groups.
- The inclusion of three diverse Latin American countries provides the possibility to explore commonalities and differences across different contexts.
- Inclusion of experience sampling methodology (ESM) enables exploration of resource-use in short-term recovery (within hours/days) and comparison with long-term recovery (over one year).
- Traditional methods of collecting data, i.e. completing questionnaires and in-depth interviews, may not be the most effective ways of engaging young people in research.
- The ongoing pandemic requires flexibility for online and offline data collection, which may increase the variance of findings and reduce the statistical power to identify differences between groups and predictor variables for outcomes.

INTRODUCTION

For peer review only - http://bmjopen.bmj.com/site/about/guidelines.xhtml

Adolescence and young adulthood are critical periods of social, behavioural and psychological maturation and change. These periods create opportunities for the development of life-long wellbeing and resilience in the face of future adversities. However, they are also associated with a significant risk for developing mental ill-health, and estimates suggest that 75% of all mental disorders begin by the age of 24[1]. The World Health Organisation (WHO) has identified improving mental health among young people as a key priority required to promote sustained economic and social development[2]. Poor mental health in the form of depression and anxiety is associated with high levels of distress and disability, future physical and psychiatric morbidity and educational and social impairment[3]. Furthermore, over half of all adolescent suicides are attributable to depression[4], making it the leading cause of mortality for this age group[5].

Urban regions, predominantly large cities, account for 81% of the population in Latin America, making it one of the most urbanised regions in the world[6]. Within these environments, people are frequently exposed to various risk factors for poor mental health. These include poverty, social fragmentation, poor education, poor housing, low employment rates, gang warfare, victimisation, violence, and widespread substance misuse[7–10].

Depression and anxiety during adolescence and youth are a particular concern within low- and middle-income countries (LMICs), where the burden of common mental disorders is greatest[2,11,12]. This includes Latin America, where young people represent one-quarter of the population. Estimated levels of depression and/or anxiety for adolescents within the region range from 17% in Colombia[13] to 26% in Argentina[14]. Those exposed to adversities such as violent conflicts, internal displacement, and poverty are at an even greater risk[15]. However, despite all the risk factors, the majority of young people do not suffer from depression and/or anxiety.

For peer review only - http://bmjopen.bmj.com/site/about/guidelines.xhtml

Due to the scarcity of financial and human resources[16], young people in deprived areas in Latin America rarely receive formal treatment when experiencing mental distress. Yet, evidence suggests that 50-60% experience symptomatic recovery within one year[17,18]. This raises the question as to which resources individuals in these contexts mobilise to prevent and overcome mental distress and build mental health resilience.

Resilience has been defined as withstanding or overcoming adversity, trauma and stress and rebounding back from distress[19]. Thus, it covers the two processes: preventing mental distress in the face of adversity and recovering from distress if and when it develops. A systematic review into the concept of resilience within mental health classified its characteristics into two components - personal and social resources[20]. Resources encompass a wide range of strengths, assets, materials, sources of information or help and means of support available to the individual. They are commonly mobilised through and in activities. These activities may be individual and reflect the health behaviours or the skills and abilities of the person[21] (personal resources); or group-based and at the community or societal level, such as captured in the concepts of social capital, social connectedness, social identity or social networks (social resources)[22]. Resources may be specific to culture, context and locality (e.g. local music group).

Previous research has focused mainly on risk factors for developing mental disorders. However, complete primary prevention of mental distress among young people, particularly in adverse urban environments, is unrealistic. This programme therefore aims to identify which personal and social resources young people use and which ones help them to overcome mental distress, reflecting the concepts of secondary and tertiary prevention[23] as well as recovery. This crucial step may lead to future interventions to reduce the burden of mental distress in young people living in deprived urban neighbourhoods in Latin America and other LMICs and help them to maintain good mental health throughout the rest of their lives.

For peer review only - http://bmjopen.bmj.com/site/about/guidelines.xhtml

Programme collaborators

This research programme is coordinated by the Unit for Social and Community Psychiatry (WHO Collaborating Centre for Mental Health Service Development) at Queen Mary University of London, and conducted in collaboration with researchers from Universidad de Buenos Aires, Pontificia Universidad Javeriana (Bogotá), Universidad Peruana Cayetano Heredia (Lima), University College London and King's College London. The research activities will take place in deprived areas of three large Latin American cities: Buenos Aires (Argentina), Bogotá (Colombia) and Lima (Peru).

A unique aspect of this programme is the partnership with well-established community-based arts organisations in each city: Fundación Crear Vale la Pena (Buenos Aires), Fundación Batuta and Familia Ayara (Bogotá), and Teatro La Plaza (Lima). Through specific art forms and creativity, these organisations work to support adolescents and young people living in vulnerable neighbourhoods to improve their quality of life, strengthen socio-emotional skills, and express and reflect on social realities and problems affecting young people. Arts-based activities are well-documented in their power to engage young people in research and to explore sensitive topics such as mental health, facilitate wider discussions and generate new knowledge[24,25]. The purpose of these collaborations is therefore to work through the arts to involve young people in this programme and its discourse about how to prevent and overcome mental distress; to explore the use of arts-based methodologies to understand resource-use in young people; and to use arts practices to disseminate the findings to a wider, non-academic audience.

Objectives

The overall aim is to identify which resources help young people living in deprived urban environments in Latin America prevent and recover from depression and/or anxiety.

The aim will be addressed via specific objectives:

For peer review only - http://bmjopen.bmj.com/site/about/guidelines.xhtml

1. Determine whether resource-use differs between adolescents and young adults with and without depression and/or anxiety,
2. Identify which resources help adolescents and young adults recover from depression and/or anxiety over a one-year period,
3. Explore existing approaches and resource-oriented interventions that are effective in preventing depression and/or anxiety in adolescents and young adults or in supporting them to recover.

METHODS AND ANALYSIS

Study design

The programme consists of several observational studies, using quantitative and qualitative methods (see Figure 1 for an overview of the study design). The methods have been finalised in a preparatory phase with workshops, focus groups and pilot interviews with young people in each of the three cities. The research activities are planned with procedures in place for both online and face-to-face data collection depending on the current local situation of the COVID-19 pandemic and government restrictions.

Figure 1: Study design

Settings

This study will take place in Buenos Aires (Argentina), Bogotá (Colombia) and Lima (Peru). In each city, a reproducible strategy was developed to sample target participants from the most deprived half of districts in each city. This involved reviewing the main indices used to collect routine statistics on the population in each setting, and using those indices which maximised comparability between settings as the basis for participant sampling. In Lima (Peru) and Bogotá (Colombia) we used the United Nations

For peer review only - http://bmjopen.bmj.com/site/about/guidelines.xhtml

Development Programme's Human Development Index[26] to assess the proportion of households in each district achieving basic living standards according to four criteria on life expectancy for health, expected years of schooling, mean years of schooling for education, and Gross National Income per capita. In Buenos Aires (Argentina), where district level statistics on the HDI were unavailable, we used the Unsatisfied Basic Needs index[27] (known as "NBI") to assess the proportion of households in each district experiencing unmet needs in at least one of five domains (housing, sanitary conditions, overcrowding, school attendance, subsistence capacity). In each setting, we ranked districts according to HDI/NBI levels and selected districts in the bottom 50% according to these scores. At district level, the HDI correlated strongly with the proportion of households in the highest SES quintile in Lima (ρ=0.84; N=49) and the proportion of households in multidimensional poverty in Bogota (ρ=0.77; N=20).

Within these communities, recruitment of the younger age group will be mostly from schools and of the older group mainly from primary care centres. Other recruitment centres might include local arts centres, youth organisations, and other community and educational organisations within defined geographical areas.

Participants

Study participants predominantly fall into two age groups: adolescents aged 15-16 years old and young adults aged 20-24 years old, with and without mental distress, living in the defined geographical areas (see above), with capacity to provide informed consent or assent alongside parental informed consent. Exclusion criteria are any severe mental illness (psychosis, bipolar disorder, schizophrenia), cognitive impairment and illiteracy (where participants are required to self-administer questionnaires).

Cross-sectional and longitudinal cohort studies

For peer review only - http://bmjopen.bmj.com/site/about/guidelines.xhtml

At each of the three sites, we will recruit 340 young people (15-16 year olds and 20-24 year olds) with mental distress and 340 young people without. Using a dimensional approach – rather than diagnostic categories – mental distress will be defined by a score of greater than nine on either the Patient Health Questionnaire-8 (PHQ-8)[28] or General Anxiety Disorder-7 (GAD-7)[29]. Both scales are self-rated. The PHQ-8 assesses symptoms of depression and the GAD-7 of anxiety. Each participant will be asked to complete the developed assessment battery.

Young people identified with mental distress will be invited to take part in the longitudinal study, which will allow comparison of resource-use in those who do and do not recover. Participants will be asked to complete a brief assessment at 6 months and the same full assessment battery at 12 months (Supplementary File 1 shows the schedule of assessments).

Experience Sampling Method study

ESM is a method of data collection which asks participants to report on their thoughts, feelings, behaviours and environment on multiple occasions as they go about their daily activities each day for a specific time period, e.g. over seven days[30]. ESM allows for a detailed assessment of the interaction between real-world context and phenomena that is unaffected by issues of recall[30]. Data will be collected using a mobile phone app called eMoodie[31], which was developed specifically for young people. The ESM study will run alongside the cross-sectional and longitudinal studies sharing the same aims and with the additional aim to determine whether young people use similar personal and social resources for short-term recovery (within hours or days) as for long-term recovery (over one year).

The ESM study will recruit 150 participants (50 in each country) already enrolled in the cross-sectional and longitudinal studies: 90 with depression and/or anxiety and 60 without, as defined above and across the two age groups (15-16 year olds and 20-24 year olds). Participants will first attend a training session with a researcher, who will explain how to use the eMoodie app, when to complete the ESM

For peer review only - http://bmjopen.bmj.com/site/about/guidelines.xhtml

assessments, to practise the questionnaires and explain each item in detail. Participants will be given phones, and/or data cards to access the internet if this is needed. Participants can contact the researcher with any questions about, or problems with, eMoodie or the ESM questions. The researcher will conduct a midweek phone call at an agreed time to discuss any concerns or problems they are experiencing with completing the questionnaires and motivate participants to complete as many ESM questionnaires as possible.

Young adult participants (20-24 years old) will complete the ESM assessment 8 times per day over 7 days, and adolescent participants (15-16 years old) will complete the ESM assessment 5 times per day for school days and 8 times per day for weekend days, again over 7 days. The times will be scheduled at random within set blocks of time. At the end of the 7-day data collection period, participants will be asked to answer a short questionnaire to briefly explore through open questions, their experience of the ESM study and any problems encountered.

The 90 participants defined with depression and/or anxiety, who participated in both the ESM study baseline and longitudinal study, will be contacted to complete the ESM assessments again after 12 months.

Case studies of good practice

This work activity will support knowledge exchange and suggest practical implications. Case studies of good practice models will illustrate and explain our previous findings. Based on the longitudinal study, we will identify and describe small-scale interventions already happening within the communities (e.g. adolescent wellbeing centres), and health and education services (e.g. school-based resilience programmes), in order to assess how communities and services can effectively prevent or respond to mental distress in young people.

Arts-based laboratories will run across 6-8 weeks by the partner arts organisations in each city to explore attitudes and perceptions about mental distress in 30 participants from the longitudinal study

For peer review only - http://bmjopen.bmj.com/site/about/guidelines.xhtml

(10 in each city) in order to reveal individual, social and contextual resilience factors and the significance of different personal and social resources in their own recovery from depression and/or anxiety. The methodologies developed in the preparatory work for researcher participation/observation will be applied at key moments throughout the workshop programme and in the discussions with young people and the arts facilitators from each organisation. Longitudinal study participants will also have the opportunity to participate in integrated arts workshops with other young people in each arts organisation.

To identify areas of good practice, 30 longitudinal study participants will be invited to take part in in-depth interviews as soon as possible after completing the 12 month follow-up. Interviews will explore which resources individuals used and found helpful or not helpful, which resources they are aware of, and any suggestions for ways in which the community or/and services could help them with their recovery. These interviews will be used alongside a stakeholder consultation workshop to recommend areas of good practice within each city, including different types of services, initiatives, projects or approaches. This workshop will be conducted with facilitated discussion asking for visual and written descriptions of existing projects and approaches. Field visits and interviews with individuals who work at and/or use these initiatives will be conducted to aid scale-up and dissemination. Finally, a stakeholder consultation will bring together the information collected to produce the case studies of each approach.

Measures

For the cross-sectional and longitudinal studies, data will be collected using a case report form (CRF). Each country-specific CRF contains the relevant language variations. A small number of questions will be varied to collect locally relevant data (socio-demographic characteristics, health insurances) and in-line with local regulations (sexual activities). All countries will be assessing the same key outcomes and resources: personal background and characteristics, social context, mental distress, quality of life,

For peer review only - http://bmjopen.bmj.com/site/about/guidelines.xhtml

For peer review only

life events, experiences and the actual use of personal resources and social resources. Supplementary File 1 summarises the questionnaires and the data collected at baseline, 6- and 12-months.

At baseline, sociodemographic data will be collected, including information about age, gender, living situation, current or obtained level of education, employment, current and previous experiences of depression and anxiety, and family history of depression and anxiety. Variables that might change over a one-year period will be collected again at the 12 month follow-up, including current main occupation and current experiences of depression and anxiety.

The ESM assessments will ask participants about their current affect, arousal state, location, company, and activity (see Supplementary File 2).

Bias

Adolescents will be primarily recruited from schools and young adults from primary care centres to ensure we recruit a varied sample of young people. Samples for both age groups will include a balance of gender. Resource-use may be affected by the presence of the pandemic and restrictions to leaving homes during lockdown, but we will assess resource-use as planned and collect information about lockdown in each city throughout data collection, to facilitate further data analysis as required.

Sample size calculations

The sample size for the cross-sectional and longitudinal studies has been calculated for identifying variables that predict recovery over a one-year period. Assuming a predictor variable (personal or social resource) exists in 10% of the population, we can detect a difference in recovery rate between 40% and 60% with 90% power and at a 5% significance level with a total sample of 762 people. Assuming a dropout rate of 25%, 1016 participants with mental distress are required at baseline.

For peer review only - http://bmjopen.bmj.com/site/about/guidelines.xhtml

Recruiting 340 people with mental distress in each of the three countries provides a total sample of 1020.

Patient and public involvement

The study is not a clinical study recruiting patients. Participants will be young people with and without mental distress in the deprived communities in Latin American cities. Young people in Bogotá, Buenos Aires and Lima were extensively involved in the preparation of the study. In arts-based workshops, focus groups and pilots of the assessment battery, they helped to develop appropriate methods for assessing resource-use. For the arts-based workshops and the arts laboratories, we are working with our partner arts organisations, which use a variety of artistic languages that can encourage engagement in research. Focus groups with adolescents (aged 15-16 years), young adults (aged 20-24 years), and professionals (mental health professionals, educators, staff from youth organisations) explored views on resources that young people use to prevent and overcome mental distress. Pilots of the assessment battery established the feasibility and acceptability of an assessment battery, including the burden of completing this through timed pilots. The findings from the preparatory work allowed us to refine the assessment measures to reflect young people's experiences, priorities and preferences. The overall research question and design of the study were informed by the literature and expertise of the multi-disciplinary research team, and patients/the public were not involved in this process.

Each research team has established a Lived Experience Advisory Panel (LEAP) comprised of young people aged 15-24 years old with lived experience of depression and/or anxiety. The purpose of the LEAPs is to engage young people and ensure their voice is present and heard throughout the research, including recruitment to and conduct of the research. The LEAP panel and our partner arts organisations will be consulted about the dissemination of findings to wider audiences of young people to continue the discourse about mental health distress.

For peer review only - http://bmjopen.bmj.com/site/about/guidelines.xhtml

Data analysis

Local researchers will transcribe verbatim the audio or video recordings from the in-depth interviews and will analyse using Framework Analysis following the stages of familiarisation; identifying a thematic framework; indexing; charting; and interpretation[32]. This will focus on common features of the identified practices and resources, which could be scaled up and more widely disseminated.

For the cross-sectional and longitudinal studies, data will be collected on paper CRFs with pseudonymised data entered onto a secure online database called REDCap, and/or participants will complete the electronic form that captures data directly onto the same database. Descriptive statistics will be reported for socio-demographic data for all participants. The numbers of individuals at each stage of the study will be reported, including the numbers potentially eligible, screened, confirmed as eligible, included, followed-up and analysed.

For the cross-sectional study, differences between the groups on each of the measures will be assessed using t-tests and chi-squared tests to determine whether resource-use differs between young people with and without depression and/or anxiety and whether the differences are apparent for the two age groups and gender.

We understand long-term recovery as recovery over a one-year period. At one-year follow up, based on the PHQ-8 and GAD-7 scores, individuals will be classified as recovered – defined as no longer screening positive for depression and/or anxiety (scoring 9 or less on either scale), or not recovered (scoring greater than 9 on either scale). The distribution and level of resources will be summarised using the median, whilst frequency and counts will summarise exposure to different community resources. A multivariate logistic regression analysis using a random effects model will assess the relationship between predictor variables and recovery, firstly for each age group, and then including the whole sample to determine whether the same resources are associated with recovery for all age groups and genders.

For peer review only - http://bmjopen.bmj.com/site/about/guidelines.xhtml

Data from the ESM assessments will be collected using the eMoodie mobile phone app, and then stored on a secure server. The data will be multilevel as multiple observations are nested within participants. Short-term recovery means any recovery within the 7-day assessment period, whether it is within hours or over the full seven days. Recovery will be assessed as a continuous variable, as improvement of any reported distress. The analysis of this will be exploratory and consider different levels of distress and improvement. Long-term recovery will use the same definition as described above. Multilevel models will assess the link between stress-related variables and negative and positive affect, controlling for potential confounding factors such as gender and sociodemographic variables. Differences between groups will be assessed, and different types of resources will be categorised and the frequency of resources within each category calculated.

ETHICS AND DISSEMINATION

For all research activities, informed consent will be obtained from participants, or assent will be obtained from participants under 18 years old alongside informed consent from their parent or legal guardian. Each research team will provide participants with information about depression and anxiety, and organisations and resources in each city for mental health support. Procedures are in place to manage any instances of risk of harm to participants or others. Where researchers conduct face-to-face data collection, they are required to follow their institutional and/or government guidelines to protect themselves and participants from COVID-19 infection. All data will be pseudonymised, handled and stored in line with the Data Protection Act 2018, General Data Protection Regulation, and national data protection laws in each partner country. Only research team members will have access to data during the study, which will be stored on REDCap, hosted on secure servers at Queen Mary, University of London. To protect the identity of participants, they will each be assigned a Participant Identification Number (PIN). The list linking participants' names to their PIN will be stored separately

For peer review only - http://bmjopen.bmj.com/site/about/guidelines.xhtml

and securely. After the completion of the programme, the datasets used and/or analysed during the cross-sectional, longitudinal and ESM studies will be available in a publically available repository. Participants' consent (or their parent/guardian's consent if under 18 years old) to share de-identified will be obtained for this purpose.

The findings will be disseminated to national stakeholders, including policymakers, professionals in healthcare and education, charities and Non-Governmental Organisations. Traditional routes of dissemination include publishing open access in peer-reviewed journals and presenting findings at national and international conferences. Through our partner arts organisations, we will also enable young people to disseminate findings to a wider, non-academic audience, including their own peer groups.

Abbreviations

LMICs- Low- and middle-income countries; WHO- World Health Organisation; ESM- Experience Sampling Method;; LEAP- Lived Experience Advisory Panel; PHQ-8- Patient Health Questionnaire-8; GAD-7- General Anxiety Disorder-7; CRF- Case Report Form; PIN- Participant Identification Number; UK- United Kingdom.

DECLARATIONS

Ethics approval

For the cross-sectional, longitudinal and ESM studies and interviews with longitudinal study participants, written approval was received:

For peer review only

For peer review only - http://bmjopen.bmj.com/site/about/guidelines.xhtml

1. From the Ethics Committee in Biomedical Research, Alberto C. Taquini Institute for Translational Medicine Research, Faculty of Medicine, University of Buenos Aires on 10/11/2020.
2. From the Faculty of Medicine - Research and Ethics Committee of the Pontificia Universidad Javeriana, Bogota on 20/11/2020 (ref: FM-CIE-1138-20)
3. From the Institutional Ethics Committee of Research of the Universidad Peruana Cayetano Heredia on 16/11/2020 (ref: 581-33-20)
4. From the Queen Mary Ethics of Research Committee on 16/11/2020 (ref: QMERC2020/02).

The study recruited its first participant in Bogotá on 26th February 2021. Data collection is yet to begin.

Data statement

The datasets used and/or analysed during the cross-sectional, longitudinal and ESM studies will be available in a publically available repository.

Acknowledgements

We would like to acknowledge our partner arts organisations for their ongoing contribution to the OLA programme: Fundación Crear Vale la Pena (Buenos Aires), Fundación Batuta and Familia Ayara (Bogotá), and Teatro La Plaza (Lima).

Author contributions

SP and VB led on the conception, design and organisation of the programme. CF and SP wrote the first draft of this manuscript. All authors contributed to the whole study design, commented on earlier drafts, and read and approved the manuscript. MS and SE particularly contributed to the statistical aspects of the programme, JBK to the method for identifying appropriate geographical areas, PH to the planning of all arts activities, NH to the adaptation of the Experience Sampling Method, CM to conceptual and methodological issues of the cohort study, RA to considerations of the Latin American

For peer review only - http://bmjopen.bmj.com/site/about/guidelines.xhtml

For peer review only

context, and CF to organisational aspects of the programme. LIB, FC, CGR, MU, and FDC specifically contributed to the adaptation of the design to the national contexts in Argentina, Colombia and Peru.

Funding

This work is supported by the Medical Research Council (grant number: MR/S03580X/1)

Competing interests

The authors declare that they have no competing interests.

For peer review only

References

1. Kessler RC, Berglund P, Demler O, et al. Lifetime prevalence and age-of-onset distributions of DSM-IV disorders in the national comorbidity survey replication. *Arch Gen Psychiatry*. 2005;62(7):768. doi:10.1001/archpsyc.62.6.593

2. Varese F, Smeets F, Drukker M, et al. Childhood adversities increase the risk of psychosis: A meta-analysis of patient-control, prospective-and cross-sectional cohort studies. *Schizophr Bull*. 2012;38(4). doi:10.1093/schbul/sbs050

3. Merikangas KR, He JP, Burstein M, et al. Lifetime prevalence of mental disorders in U.S. adolescents: Results from the national comorbidity study-adolescent supplement (NCS-A). *J Am Acad Child Adolesc Psychiatry*. 2010;49(10):980-989. doi:10.1016/j.jaac.2010.05.017

4. Thompson MP, Kingree JB, Lamis D. Associations of adverse childhood experiences and suicidal behaviors in adulthood in a U.S. nationally representative sample. *Child Care Health Dev*. 2019;45(1):121-128. doi:10.1111/cch.12617

5. Horn AB, Cañizares C, Gómez Y. Prevention of adolescent depression in the Spanish-speaking world. *Int J Environ Res Public Health*. 2014;11(6):5665-5683. doi:10.3390/ijerph110605665

For peer review only - http://bmjopen.bmj.com/site/about/guidelines.xhtml

6. United Nations, Department of Economic and Social Affairs PD. *World Urbanization Prospects 2018: Highlights*.; 2019. doi:10.18356/6255ead2-en

7. Patel V, Flisher AJ, Hetrick S, et al. Mental health of young people: a global public-health challenge. *Lancet*. 2007;369(9569):1302-1313. doi:10.1016/S0140-6736(07)60368-7

8. Kim D. Blues from the neighborhood? Neighborhood characteristics and depression. *Epidemiol Rev*. 2008;30(1). doi:10.1093/epirev/mxn009

9. Morgan C, Reininghaus U, Reichenberg A, et al. Adversity, cannabis use and psychotic experiences: Evidence of cumulative and synergistic effects. *Br J Psychiatry*. 2014;204(5). doi:10.1192/bjp.bp.113.134452

10. Thapar A, Collishaw S, Pine DS, et al. Depression in adolescence. *Lancet*. 2012;379(9820). doi:10.1016/S0140-6736(11)60871-4

11. Zammit S, Lewis G, Dalman C, et al. Examining interactions between risk factors for psychosis. *Br J Psychiatry*. 2010;197(3):207-211. doi:10.1192/bjp.bp.109.070904

12. Ferrari AJ, Charlson FJ, Norman RE, et al. Burden of Depressive Disorders by Country, Sex, Age, and Year: Findings from the Global Burden of Disease Study 2010. *PLoS Med*. 2013;10(11). doi:10.1371/journal.pmed.1001547

13. Murcia EH, Losada YL, Rojas LA, et al. Prevalencia de la depresión infantil en neiva (Colombia). *Av en Psicol Latinoam*. 2009;27(1).

14. Méndez X, Rosa AI, Montoya M, et al. Tratamiento psicológico de la depresión infantil y adolescente: ¿Evidencia o promesa? *Psicol Conductual*. 2002;10(3).

15. Navarro L, Moscoso J, De La Cruz G. La investigación de la depresión en adolescentes en el Perú: una revisión sistemática. *Lib Rev Peru Psicol*. 2017;23(1). doi:10.24265/liberabit.2017.v23n1.04

For peer review only

For peer review only - http://bmjopen.bmj.com/site/about/guidelines.xhtml

16. Rathod S, Pinninti N, Irfan M, et al. Mental Health Service Provision in Low- and Middle-Income Countries. *Heal Serv Insights*. 2017;10:1-7. doi:10.1177/1178632917694350

17. March JS, Silva S, Petrycki S. Fluoxetine, cognitive-behavioral therapy, and their combination for adolescents with depression: Treatment for Adolescents with Depression Study (TADS) randomized controlled trial. *J Am Med Assoc*. 2004;292(7):807-820. doi:10.1001/jama.292.7.807

18. Stapinski LA, Montgomery AA, Heron J, et al. Depression Symptom Trajectories and Associated Risk Factors among Adolescents in Chile. *PLoS One*. 2013;8(10). doi:10.1371/journal.pone.0078323

19. Anderson K, Priebe S. Concepts of resilience in adolescent mental health research. *J Adolesc Heal*. in press.

20. Gross JJ, John OP. Individual Differences in Two Emotion Regulation Processes: Implications for Affect, Relationships, and Well-Being. *J Pers Soc Psychol*. 2003;85(2):348-362. doi:10.1037/0022-3514.85.2.348

21. Pargas RCM, Brennan PA, Hammen C, et al. Resilience to Maternal Depression in Young Adulthood. *Dev Psychol*. 2010;46(4):805-814. doi:10.1037/a0019817

22. Kim J. Neighborhood disadvantage and mental health: The role of neighborhood disorder and social relationships. *Soc Sci Res*. 2010;39(2):260-271. doi:10.1016/j.ssresearch.2009.08.007

23. Simeonsson RJ. Primary, Secondary, and Tertiary Prevention in Early Intervention. *J Early Interv*. 1991;15(2). doi:10.1177/105381519101500202

24. Fenge LA, Hodges C, Cutts W. Seen But Seldom Heard: Creative Participatory Methods in a Study of Youth and Risk. *Int J Qual Methods*. 2011;10(4). doi:10.1177/160940691101000408

25. Yonas MA, Burke JG, Miller E. Visual Voices: A Participatory Method for Engaging Adolescents

For peer review only - http://bmjopen.bmj.com/site/about/guidelines.xhtml

in Research and Knowledge Transfer. *Clin Transl Sci*. 2013;6(1). doi:10.1111/cts.12028

26. United Nations Development Programme Human Development Reports. Accessed January 13, 2021. http://hdr.undp.org/en/content/human-development-index-hdi

27. Santos ME. *Measuring Multidimensional Poverty in Latin America: Previous Experience and the Way Forward*.; 2014.

28. Kroenke K, Strine TW, Spitzer RL, et al. The PHQ-8 as a measure of current depression in the general population. *J Affect Disord*. 2009;114(1-3). doi:10.1016/j.jad.2008.06.026

29. Spitzer RL, Kroenke K, Williams JBW, et al. A brief measure for assessing generalized anxiety disorder: The GAD-7. *Arch Intern Med*. 2006;166(10). doi:10.1001/archinte.166.10.1092

30. Myin-Germeys I, Kasanova Z, Vaessen T, et al. Experience sampling methodology in mental health research: new insights and technical developments. *World Psychiatry*. 2018;17(2):123-132. doi:10.1002/wps.20513

31. Domoff SE, Banga CA, Borgen AL, et al. Use of passive sensing to quantify adolescent mobile device usage: Feasibility, acceptability, and preliminary validation of the eMoodie application. *Hum Behav Emerg Technol*. Published online 2021. doi:10.1002/hbe2.247

32. Richie J, Spencer L. Qualitative data analysis for applied policy research. In A. Bryman & R.G. Burgess (Eds). In: *Analyzing Qualitative Data*. ; 1994.

For peer review only

For peer review only - http://bmjopen.bmj.com/site/about/guidelines.xhtml

Figure 1: Study design

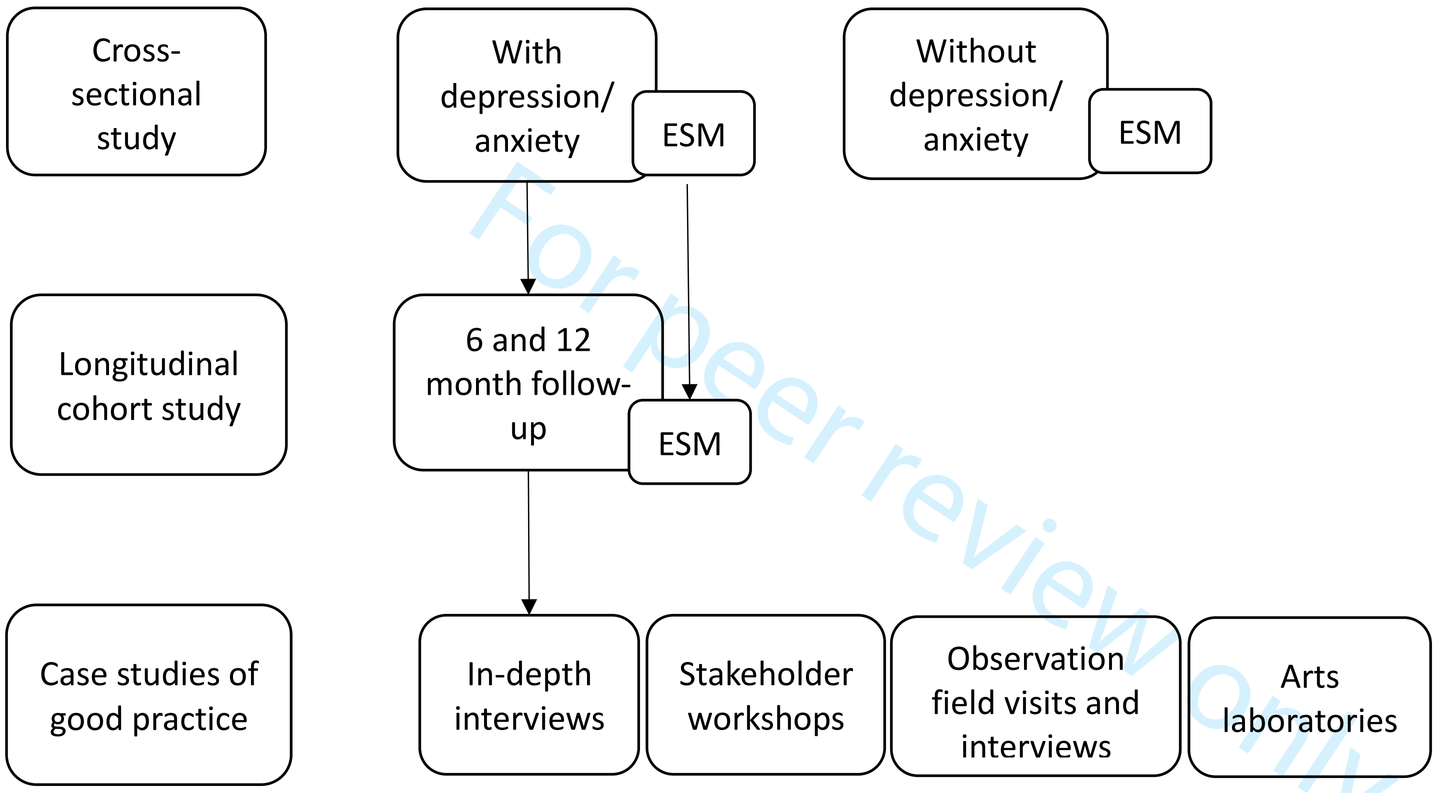

For peer review only

Supplementary File 1: Schedule of assessments for the cross-sectional and longitudinal studies

Item measured	Scale/ assessment method	Description of scale	Baseline	6-month follow-up	12-month follow-up
**DEMOGRAPHICS**					
Demographics	Current living situation	Includes variables like gender, age, education level etc	X		X*
	History of depression and anxiety	Participants' and their parents' experiences of mental distress and treatment received	X		X*
**MEASURES OF MENTAL DISTRESS**					
Degree of distress	Patient Health Questionnaire-8 (PHQ-8)	Measures degree of experiencing a list of symptoms associated with depression	X	X	X
	Generalised Anxiety Disorder-7 (GAD-7)	Measures degree of experiencing a list of symptoms associated with anxiety	X	X	X
Drug use	The Alcohol, Smoking and Substance Involvement Screening Test (ASSIST) - adapted	Measures drug use in the lifetime and last 3 months	X		X
	Adapted Teen Addiction Severity Index (T-ASI) - adapted	Measures drug use including alcohol use and severity	X		X
Life events	Adolescent appropriate life events scale - adapted	Captures experience of life events in their lifetime and in the last year or six month respectively	X	X	X
**MEASURES OF RESOURCES**					
General resources	Open question	What participants do when they feel mentally distressed	X		X
Impact of COVID-19	Closed questions	Brief assessment of how COVID-19 has impacted on various activities	X	TBC	TBC
**MEASURES OF PERSONAL RESOURCES**					
Quality of life	Manchester Short Assessment of Quality of Life (MANSA)	Measures perception of how satisfied they are with different aspects of their lives	X		X
Sex life	Closed question	Whether they have had sex with another person in the last month	X		X
Coping style	Child's Coping Strategy Checklist	Measures how individuals deal with problems and stress	X		X
Resilience	Connor-Davidson Resilience Scale (CD-RISC 10)	Measures resilience in response to stressful events, tragedy or trauma	X		X
**MEASURES OF SOCIAL RESOURCES**					
Use of healthcare and other services	Client Service Receipt Inventory (CSRI) - adapted	Measures frequency of use of healthcare and social services	X	X	X
Social support	Scale of Perceived Social Support	Measures perception of social support network	X		X

For peer review only

Social capital	Adapted Social Capital Assessment Tool (ASCAT) - adapted	Asks about perceptions of and engagement with community groups	X		X
Family support	Open questions	Asks which family member they speak to about feelings and emotions, and which family members speak to them about feelings and emotions	X		X
**MEASURES OF ACTIVITIES**					
Sports activity	Open/closed questions	Asks about sports activities including frequency of participation and the nature of these activities	X		X
Arts activity	Open/closed questions	Asks about arts activities including frequency of participation and the nature of these activities	X		X
Internet use	Question 59 from REACH study - adapted	Measures internet use via agreement with a list of statements	X		X

Legend:
X: questionnaire included
X: various items included (e.g. only sociodemographic items that might have changed will be included)*
TBC: to be confirmed depending on the COVID-19 situation in partner countries

For peer review only

Supplementary File 2: Summary of questions that form the ESM assessment

Item measured	Question	Description
Location	Where are you now?	1 item asking participants to state their location. Participants choose from predefined categories or select "other" and provide more detail
Activity	What are you doing now?<br>Time spent doing this activity since last "beep" (notification)?	1 open question asking participants what they are doing (main activity)<br>1 item asking the participants to indicate time spent doing this activity since the last notification
Company	Who are you doing this activity with?	1 item asking participants to state who they are doing their main activity with. Participants choose from predefined categories or select "other" and provide more detail.
Affect	How are you feeling now?	1 item asks participants to rate how they are feeling on a 7-point Likert scale ranging from 1 ("extremely unhappy") to 7 ("extremely happy")
Arousal	How nervous or relaxed are you now?	1 item asking participants to rate how nervous/relaxed they are feeling on a 7-point Likert scale ranging from 1 "very nervous" to 7 "very relaxed"

For peer review only

STROBE 2007 (v4) checklist of items to be included in reports of observational studies in epidemiology*
Checklist for cohort, case-control, and cross-sectional studies (combined)

Section/Topic	Item #	Recommendation	Reported on page #
**Title and abstract**	1	(*a*) Indicate the study's design with a commonly used term in the title or the abstract	2
		(*b*) Provide in the abstract an informative and balanced summary of what was done and what was found	2,3
**Introduction**			
Background/rationale	2	Explain the scientific background and rationale for the investigation being reported	4,5
Objectives	3	State specific objectives, including any pre-specified hypotheses	6,7
**Methods**			
Study design	4	Present key elements of study design early in the paper	7
Setting	5	Describe the setting, locations, and relevant dates, including periods of recruitment, exposure, follow-up, and data collection	7-10
Participants	6	(*a*) *Cohort study*—Give the eligibility criteria, and the sources and methods of selection of participants. Describe methods of follow-up<br>*Case-control study*—Give the eligibility criteria, and the sources and methods of case ascertainment and control selection. Give the rationale for the choice of cases and controls<br>*Cross-sectional study*—Give the eligibility criteria, and the sources and methods of selection of participants	8
		(*b*) *Cohort study*—For matched studies, give matching criteria and number of exposed and unexposed<br>*Case-control study*—For matched studies, give matching criteria and the number of controls per case	
Variables	7	Clearly define all outcomes, exposures, predictors, potential confounders, and effect modifiers. Give diagnostic criteria, if applicable	11
Data sources/ measurement	8*	For each variable of interest, give sources of data and details of methods of assessment (measurement). Describe comparability of assessment methods if there is more than one group	Additional File 2
Bias	9	Describe any efforts to address potential sources of bias	12
Study size	10	Explain how the study size was arrived at	11, 12
Quantitative variables	11	Explain how quantitative variables were handled in the analyses. If applicable, describe which groupings were chosen and why	14
Statistical methods	12	(*a*) Describe all statistical methods, including those used to control for confounding	13, 14
		(*b*) Describe any methods used to examine subgroups and interactions	14
		(*c*) Explain how missing data were addressed	
		(*d*) *Cohort study*—If applicable, explain how loss to follow-up was addressed<br>*Case-control study*—If applicable, explain how matching of cases and controls was addressed	

For peer review only

		Cross-sectional study—If applicable, describe analytical methods taking account of sampling strategy	
		(*e*) Describe any sensitivity analyses	
**Results**			Section is N/A
Participants	13*	(a) Report numbers of individuals at each stage of study—eg numbers potentially eligible, examined for eligibility, confirmed eligible, included in the study, completing follow-up, and analysed	
		(b) Give reasons for non-participation at each stage	
		(c) Consider use of a flow diagram	
Descriptive data	14*	(a) Give characteristics of study participants (eg demographic, clinical, social) and information on exposures and potential confounders	
		(b) Indicate number of participants with missing data for each variable of interest	
		(c) *Cohort study*—Summarise follow-up time (eg, average and total amount)	
Outcome data	15*	*Cohort study*—Report numbers of outcome events or summary measures over time	
		Case-control study—Report numbers in each exposure category, or summary measures of exposure	
		Cross-sectional study—Report numbers of outcome events or summary measures	
Main results	16	(*a*) Give unadjusted estimates and, if applicable, confounder-adjusted estimates and their precision (eg, 95% confidence interval). Make clear which confounders were adjusted for and why they were included	
		(*b*) Report category boundaries when continuous variables were categorized	
		(*c*) If relevant, consider translating estimates of relative risk into absolute risk for a meaningful time period	
Other analyses	17	Report other analyses done—eg analyses of subgroups and interactions, and sensitivity analyses	
**Discussion**			Section is N/A
Key results	18	Summarise key results with reference to study objectives	
Limitations	19	Discuss limitations of the study, taking into account sources of potential bias or imprecision. Discuss both direction and magnitude of any potential bias	
Interpretation	20	Give a cautious overall interpretation of results considering objectives, limitations, multiplicity of analyses, results from similar studies, and other relevant evidence	
Generalisability	21	Discuss the generalisability (external validity) of the study results	
**Other information**			
Funding	22	Give the source of funding and the role of the funders for the present study and, if applicable, for the original study on which the present article is based	18

*Give information separately for cases and controls in case-control studies and, if applicable, for exposed and unexposed groups in cohort and cross-sectional studies.

Note: An Explanation and Elaboration article discusses each checklist item and gives methodological background and published examples of transparent reporting. The STROBE checklist is best used in conjunction with this article (freely available on the Web sites of PLoS Medicine at http://www.plosmedicine.org/, Annals of Internal Medicine at http://www.annals.org/, and Epidemiology at http://www.epidem.com/). Information on the STROBE Initiative is available at www.strobe-statement.org.

For peer review only
